# A Qualitative Comparison of the Post-COVID Retirement Experience Between Japanese and American Older Adults

**DOI:** 10.1093/geroni/igab046.1745

**Published:** 2021-12-17

**Authors:** Dana Bradley, Mariko Nishikitani, Mie Ariyoshi, Fumihiko Yokota, Gretchen Tucker, Laura Allen

**Affiliations:** 1 UMBC Erickson School of Aging Studies, Baltimore, Maryland, United States; 2 Kyushu University Hospital, Medical Information Center, Kyushu University Hospital, Fukuoka, Japan; 3 St Cahterines University, Matsuyama, Ehime, Japan; 4 Kyushu University, Kyushu, Fukuoka, Japan; 5 Bar-Ilan University, Ramat Gat, Tel Aviv, Israel

## Abstract

The purpose of this collaborative study between researchers in Japan and the U.S. was to understand the retirement experience and potential changes in social interactions (amount, type, and mode of communication) among older adults living independently in the community. Specifically, we were interested in individuals’ expectations about retirement and the types of social interactions experienced prior to and post-retirement, situated within the context of the COVID-19 pandemic. Both research teams conducted in-depth one-on-one interviews with community-residing retired older adults in early 2021. In the findings we explore similarities and differences between the retirement experiences of Japanese and American older adults, including methodological differences that transpired; specifically, we evaluate the meaning and experience of the COVID-19 pandemic in each of these cultural contexts and how retired older adults experienced its impact in their social interactions.

